# First episode psychosis and beyond: much progress made but much more needed

**DOI:** 10.1186/s12888-023-04639-6

**Published:** 2023-03-07

**Authors:** Shuichi Suetani, Wei Wang

**Affiliations:** 1 Institute for Urban Indigenous Health, Windsor, QLD Australia; 2 Physical health and mental health stream, Queensland Centre for Mental Health Research, Wacol, QLD Australia; 3 Queensland Brain Institute, The University of Queensland, St Lucia, QLD Australia; 4 School of Medicine and Dentistry, Griffith University, Southport, QLD Australia; 5 Norwegian University of Science and Technology, Trondheim, Norway

## Abstract

First episode psychosis remains one of the most critical research areas in psychiatry. Much progress has been made, but more progress is required to translate the ideas and promises into reality. In this Editorial, we provide the context and invite contributions for our *BMC Psychiatry Collection* on First Episode Psychosis.

In the 1980s, a 10-bed clinical research unit for people with first episode psychosis (FEP) was established in Melbourne, Australia, with two main tasks [1]. First, to reduce and prevent the harm that people with FEP were exposed to by separating them from people with more pervasive mental disorders and the messages and treatments that were draining hope and optimism. Second, to develop and evaluate psychosocial interventions for patients and their families that were relevant and appropriate for the stage of illness and psychosocial development. The intriguing idea of a critical period for psychotic disorders – perhaps the first five years after the onset of FEP – having a disproportionate long-term impact was proposed [2]. Australia led the innovation by establishing EPPIC (Early Psychosis Prevention and Intervention Centre), and other countries soon followed with similar programs. Much like another Australian innovation, Lithium [3], the FEP paradigm (“*a stitch in time…*”) changed how we conceptualize and practice psychiatry. Key concepts such as the duration of untreated psychosis [4], prodromal symptoms [5], and ultra-high risk mental state [6] became well embedded within our lexicon. The International Early Psychosis Association was established in the early 1990s, and *Early Intervention in Psychiatry* journal was published in the late 2000s [1].

Despite its promises and intuitive logic behind the idea, the FEP paradigm also attracted much criticism. In 2011, Professor Allen Frances, the Chair of the task force overseeing the development and revision of the fourth edition of the Diagnostic and Statistical Manual of Mental Disorders, expressed his concern that the FEP paradigm was prematurely being implemented into clinical practice without sufficient foundations. Namely, he argued (i) accurate diagnosis, (ii) interventions with proven efficacy and (iii) proven safety had not yet been established adequately for the FEP paradigm [7]. More than a decade has passed but the detection and diagnosis of FEP remain challenging due to its fluid, heterogeneous and often vague symptomatology [8]. The stigma associated with mental illness, particularly psychotic disorders, persists, making timely access and diagnosis difficult [9]. Further, only around one in four people presenting with a high clinical risk for psychosis actually transition into psychosis within three years [10]. To this day, there remain no approved interventions that have been shown to improve the long-term course of psychotic disorders, and specialized integrated early intervention services do not substantially reduce the risk of having a relapse compared to standard care [11]. More recently, this high psychosis risk cum psychotic disorder transition concept has been questioned. Van Os and Guloksuz [12] argue that it is neither clinically useful nor scientifically valid to reduce the transdiagnostic expression of psychosis in early states of multidimensional psychopathology to the binary concepts of risk and transition. Accordingly, some groups, including Orygen in Australia, are moving towards widening the “at risk mental state” model beyond psychotic disorders to take a more transdiagnostic approach [13]. Heated debates around the benefits of the FEP paradigm in the real world persist even (or perhaps *especially*) in Australia, where Professor Patrick McGorry was awarded the Australian of the Year in 2010 for his work in youth mental health [14–17]. Patel captured one sentiment among the clinicians at the coal face in the response to an article titled *Why do psychiatrists doubt the value of early intervention? The power of illusion* by McGorry and Mei [17]: “Clinician’s reality may reflect the experience of seeing the long-term outcomes of schizophrenia unchanged by [early intervention] whilst the authors reality reflects the experience of shorter-term outcomes for a much broader and heterogeneous psychosis group. The progressive underfunding of services for the former during a period of rapid growth in services for an even more ill-defined ‘youth mental health’ group championed by McGorry further diminishes his relevance of his views for the ‘disillusioned’ clinician” [18].

Forty years on, some critical questions remain unanswered in the field of FEP research. First, what are the causes of FEP? Is the onset of FEP one of the earliest signs of a pervasive brain disease, or do cultural, social and environmental factors contribute to FEP? Related to this, is the onset of FEP really the beginning of a critical period for intervention [2]? If so, how long does this critical period last and what more can we do to reduce the duration of untreated psychosis? Second, what are the consequences of FEP? What are the different phenotypes and endotypes of FEP? What are the long-term functional and personal impacts of FEP beyond clinical prognosis? Can we “cure” FEP? If not, why not? Finally, and perhaps most importantly, how do we translate the short-term efficacy seen in clinical trials into sustained effectiveness for our patients and their families in real life? Is there more we can do at the individual level? What more should we be doing at the societal and policy levels? The truth is, despite much progress in the field, the mortality gap among people with severe mental disorders has widened [19], and the recovery rate among people with psychotic disorders remains unchanged [20].

The FEP paradigm has given us a unique and valuable foundation to explore and examine mental disorders in general, psychotic disorders in particular. We have come a long way, but we are not there yet. We hope this Collection will provide another step towards a better future for people experiencing FEP and their families. We progress by testing ideas and rejecting ideologies. We must continue to seek effective and safe pharmacological, psychosocial and other interventions that will reduce and resolve the sufferings associated with FEP.

## Data Availability

Not applicable.
